# Research priorities in deceptive online gambling platform design research: We need to understand behavioral usage patterns in order to inform safer platform design

**DOI:** 10.1111/add.70176

**Published:** 2025-08-13

**Authors:** Philip Newall

**Affiliations:** ^1^ School of Psychological Science University of Bristol Bristol UK

**Keywords:** behavioral design, behavioral science, dark nudges, dark patterns, evidence, sludge

*Existing research on online gambling platform design has been largely limited to observational studies, which cannot help us to understand usage or harm. The responses to the target article have helped me to highlight some of the promising directions that should be explored to solve these research gaps*.

In my article, I argued that online gambling platforms are often designed in deceptive ways to maximize the amount of time and money that people spend on them [1], as a digital extension of Schüll's research on the deceptive design of land‐based casinos [2]. This is important given online gambling's increasing ascendance internationally [3] and can also be seen as an extension to research on the harmful structural characteristics of many gambling products [4]. My article arranges existing literature on this topic [1], and further argues that three factors have inhibited this research area: the existence of competing terms such as sludge [5], dark patterns [6] and dark nudges [7]; a preponderance of grey literature; and a lack of access to behavioral data. This last factor is the most critical, and in responding to these commentaries, I argue optimistically that multiple routes are open to facilitate data access to promote an understanding of how people use online gambling platforms, to inform regulation to promote safer design.

Clark and Weston's [8] commentary argues that these issues are important in the North American context, where 30 United States states have legalized online sports betting, and one Canadian province has introduced a competitive online gambling marketplace, with another province being set to follow. The Canadian changes disrupt a stable status quo, where gambling was hitherto allowed only under provincial state‐owned monopolies [9]. Although concerning, this rapid yet uneven spread could, if sufficient high‐quality data exist, serve as the closest possible equivalent to a real‐world controlled experiment. Furthermore, state‐owned gambling operators are more willing to collaborate in research than privately owned operators [10]. This willingness should be harnessed by researchers with strong relationships with state‐owned operators, by requesting high‐resolution data on patterns of platform use.

Field and Gaskell's [11] commentary is largely supportive, arguing that neurocognitive models of gambling harm place too much emphasis on the person compared to the product. They further argue that the unique features of online gambling might require making alterations to evidence‐based treatments such as cognitive behavioral therapy. I fully agree with these points, and contend that further restrictions on the speed of online gambling, to more closely resemble that of land‐based gambling, might also logically follow from them [12]. While gambling policymakers often say that there is ‘no evidence’ to support harm‐prevention policies, there is also often no evidence to support the status quo—on many topics there is simply no evidence [13]. United Kingdom‐based policymakers should, therefore, consider the evidence‐building benefits of improved independent data infrastructure [14], with the Nordic countries having one model worth following [15]. Individuals could also be facilitated to share their own data directly with researchers [16]. Under any approach, solving these issues will require a broad range of stakeholder collaboration [17, 18].

In conclusion, I remain hopeful that among these many paths, that some will prove fruitful in terms of improving our understanding of how people use online gambling platforms, to inform regulation to promote safer design.

## AUTHOR CONTRIBUTIONS

**Philip Newall:** Conceptualization.

## DECLARATION OF INTERESTS

P.N, is a member of the Advisory Board for Safer Gambling—an advisory group of the Gambling Commission in Great Britain. In the last 3 years, P.N. has contributed to research projects funded by the Academic Forum for the Study of Gambling, Alberta Gambling Research Institute, BA/Leverhulme, Canadian Institute for Health Research, Clean Up Gambling, Gambling Research Australia and the Victorian Responsible Gambling Foundation. P.N. has received honoraria for reviewing from the Academic Forum for the Study of Gambling and the Belgium Ministry of Justice, travel and accommodation funding from the Alberta Gambling Research Institute and the Economic and Social Research Institute and open access fee funding from the Academic Forum for the Study of Gambling and Greo Evidence Insights.
